# Comparable Benefits in Heart Failure Hospitalization and Survival with Sacubitril/Valsartan Therapy in CRT Nonresponders and HFrEF Patients Without CRT Indication †

**DOI:** 10.3390/jcm14176098

**Published:** 2025-08-28

**Authors:** Krisztina Mária Szabó, Anna Tóth, László Nagy, László Tibor Nagy, Gábor Sándorfi, Marcell Clemens, Attila Csaba Nagy, Arnold Péter Ráduly, Attila Borbély, Judit Barta, Zoltán Csanádi

**Affiliations:** 1 Department of Cardiology, University of Debrecen, 4032 Debrecen, Hungary; toth.anna@med.unideb.hu (A.T.); dr.nagy.laszlo@med.unideb.hu (L.N.); nagy.laszlo@med.unideb.hu (L.T.N.); sandorfi.gabor@med.unideb.hu (G.S.); clemens.marcell@med.unideb.hu (M.C.); raduly.arnold@med.unideb.hu (A.P.R.); borbelya@med.unideb.hu (A.B.); barta.judit@med.unideb.hu (J.B.); csanadi.zoltan@med.unideb.hu (Z.C.); 2 Department of Health Informatics, Faculty of Health Sciences, University of Debrecen, 4032 Debrecen, Hungary; attilanagy@med.unideb.hu

**Keywords:** heart failure with reduced ejection fraction, cardiac resynchronization therapy, sacubitril/valsartan, outcomes

## Abstract

**Background:** Sacubitril/valsartan (S/V) improves left ventricular (LV) function and clinical outcome in heart failure (HF) with reduced ejection fraction (HFrEF). Data on its clinical value in the specific cohort of HFrEF patients demonstrating no adequate response to cardiac resynchronization therapy (CRT nonresponders; CRT-NRs) are limited. Herein, we investigated the impact of S/V initiated as a replacement for ACEi/ARB therapy in CRT nonresponder (CRT-NR) patients. **Methods:** Our HF database was searched to identify CRT-NRs who received S/V treatment for at least 6 months as a replacement for ACEi/ARB (Group I; 70 patients) and CRT-NRs who remained on ACEi/ARB (Group II, 70). In addition, HFrEF patients without CRT indication who received S/V therapy for at least 6 months (Group III; 135) were also included in this analysis. The primary endpoint was the composite of all-cause mortality including heart transplantation (HTx) or left ventricular assist device implantation (LVAD) and HF hospitalization (HFH). Secondary endpoints were (i) all-cause mortality+HTx+LVAD and (ii) HFH analyzed separately. **Results:** Over a median follow-up of 22 months, the primary composite endpoint occurred in 27 out of 70 patients (38.57%) in Group I, 43 out of 70 patients (61.42%) in Group II, and 60 out of 135 patients (44.42%) in Group III. The differences were significant between Groups I and II (*p*: 0.005), as well as between Group II and III (*p*: 0.012), while the two groups on S/V (Group I and III) demonstrated similar outcomes (*p* = 0.465). HFH analyzed separately as a secondary endpoint occurred in 19 out of 70 patients (27.14%) in Group I, 38 out of 70 patients (54.28%) in Group II, and 36 out of 135 patients (26.66%) in Group III (Group I vs. II *p*: 0.001; Groups II vs. III *p*: 0.001, Group I vs. III, *p*: 0.896). All-cause mortality+HTx+LVAD analyzed separately as the other secondary endpoint demonstrated no significant differences among the three groups. **Conclusions:** S/V therapy improved HFH but not mortality in CRT-NR patients. Comparable improvement was demonstrated after SV in the CRT-NR and in the general HFrEF cohort with no CRT indication.

## 1. Introduction

Cardiac resynchronization therapy (CRT) is recommended for patients who experience symptomatic heart failure (HF) despite evidence-based HF treatment presenting with reduced ejection fraction (HFrEF) and a wide QRS complex to alleviate symptoms and reduce morbidity and mortality [1]. Improvement in HF symptoms and especially in long-term survival have consistently been linked to reverse remodeling measured 6–12 months after CRT. However, approximately 30–40% of patients receiving CRT treatment show no significant change in echocardiographic parameters and in biomarkers related to HF after device implantation [2,3,4].

Optimized medical therapy in line with guideline recommendations for at least 3 months is required before considering CRT implantation. According to recent HF guidelines this include renin angiotensin aldosterone system (RAAS) inhibitors, beta blockers (BB), mineralocorticoid receptor antagonists (MRA), and sodium glucose cotransporter 2 (SGLT2) inhibitors in all symptomatic HFrEF patients [5,6,7]. As far as RAAS inhibition is concerned, according to the European guidelines, angiotensin-converting enzyme inhibitors (ACEi) or angiotensin II receptor blockers (ARBs) are considered as a first-line choice to be replaced with angiotensin receptor neprilysin inhibitor (ARNI) in patients demonstrating no improvement in their HF status, while the American organizations (ACC/AHA) recommend ARNI as the first-line treatment in all HFrEF patients [6,7]. These recommendations were based on the results of the PARADIGM-HF trial, which demonstrated that S/V was superior to ACEi/ARB in lowering mortality rates and reducing heart failure hospitalization (HFH) [8,9,10]. 

While the significance of medical therapy optimization before CRT implantation has been emphasized, data on the “fine tuning” of medical treatment after device implantation are scarce [11,12]. 

Our group recently demonstrated that in patients showing no improvement on ACE2i or on ARB therapy at least 6 months after CRT implantation, S/V utilized as a substitute for ACEi/ARB induced left ventricular reverse remodeling, demonstrated by an increase in left ventricular ejection fraction (LV EF) and decreases in both left ventricular end-systolic diameter (LV ESD) and plasma N-terminal pro-B-type natriuretic peptide (NT-proBNP) concentration. Further, the magnitude of improvement observed was similar to what was measured after the initiation of S/V in general HFrEF patients who had no indication for CRT [13].

Herein, we report on the long-term outcomes of CRT nonresponder (CRT-NR) patients who were initiated on S/V as a replacement for ACEI/ARB therapy versus those who were not. Patients with HFrEF without CRT indication started on S/V medication were also included in this research.

## 2. Materials and Methods

### 2.1. Study Design and Patients

The study design has been reported in detail [13]. Briefly, the clinical database of our department was searched between January 2018 and June 2021 to identify HFrEF patients with LV EF < 40% receiving guideline-dictated medical therapy including ACEi/ARB or S/V. HFrEF patients implanted with a CRT pacemaker (CRT-P) or a CRT defibrillator (CRT-D), as well as those with no CRT indication, were screened. Patients with CRT who had a biventricular capture rate of less than 95% were excluded from further analysis. LV EF values measured with transthoracic echocardiography using Simpson’s biplane method at baseline and 6 months after CRT implantation were compared, and patients demonstrating at least a 10% increase were categorized as responders (CRT-Rs) while those with no or less than 10% improvement were categorized as CRT-NRs. Only CRT-NR patients were included in this study, and they were subcategorized according to the type of RAAS inhibition they received: patients who remained on ACEi/ARB throughout the study period or those who were switched to S/V as a replacement of ACEi/ARB. Additionally, HFrEF patients who had no indication for CRT but were initiated on S/V throughout the course of the study were also included in the analysis. Patients were assigned to any of the 3 groups based on the following criteria:

*Group I: CRT-NR patients initiated on S/V.* No or less than 10% improvement in LV EF was demonstrated 6 months after CRT on optimized medical therapy including ACEi/ARB. S/V was initiated after termination of ACEi/ARB therapy, and the results of 2D echocardiography and plasma NT-proBNP levels were obtained both before the initiation of S/V and for a minimum 6 months afterwards.

*Group II: CRT-NR on ACEi/ARB.* No or less than 10% improvement in LV EF was demonstrated 6 months after CRT. Patients were receiving ACEi/ARB treatment throughout the entire study period, and the results of 2D echocardiography and plasma NT-proBNP concentrations were available from two time points with a minimum interval of 6 months between them.

*Group III: HFrEF patients initiated on S/V therapy*. These patients represented a general cohort of HFrEF with no indication for CRT implantation based on LV EF and QRS duration criteria. ACEi/ARB therapy was stopped, and S/V initiated, during the study period, and the results of 2D echocardiography and plasma NT-proBNP levels obtained prior to the initiation of S/V and at least 6 months afterwards were available.

Physical exam, 12-lead ECG, 2D echocardiography, and laboratory tests were performed routinely at each follow-up visit. Serum NT-proBNP concentrations were determined using the Elecsys proBNP II assay (catalog number 09315268160; Roche Diagnostics, Mannheim, Germany) on a Cobas e 411 analyzer, employing an electrochemiluminescence immunoassay according to the manufacturer’s instructions.

The study was approved by the Scientific and Research Ethics Committee (Local Ethics Committee-ETT TUKAB) of the University of Debrecen. File no: BMEÜ/4388- 1/2022/EKU.

### 2.2. Endpoints of Patient Outcomes

The initial date of follow-up was the day when patients in Groups I and III were initiated on S/V. As patients in Group II were kept on continuous ACEi/ARB therapy, the starting time of follow-up was considered the date of the 2nd echocardiography at least 6 months after CRT implantation, which was utilized to categorize a patient as a nonresponder. During follow-up, the events collected were death from any cause, heart transplantation (HTx), the need for left ventricular assist device (LVAD) implantation, and HFH.

**Figure 1:**
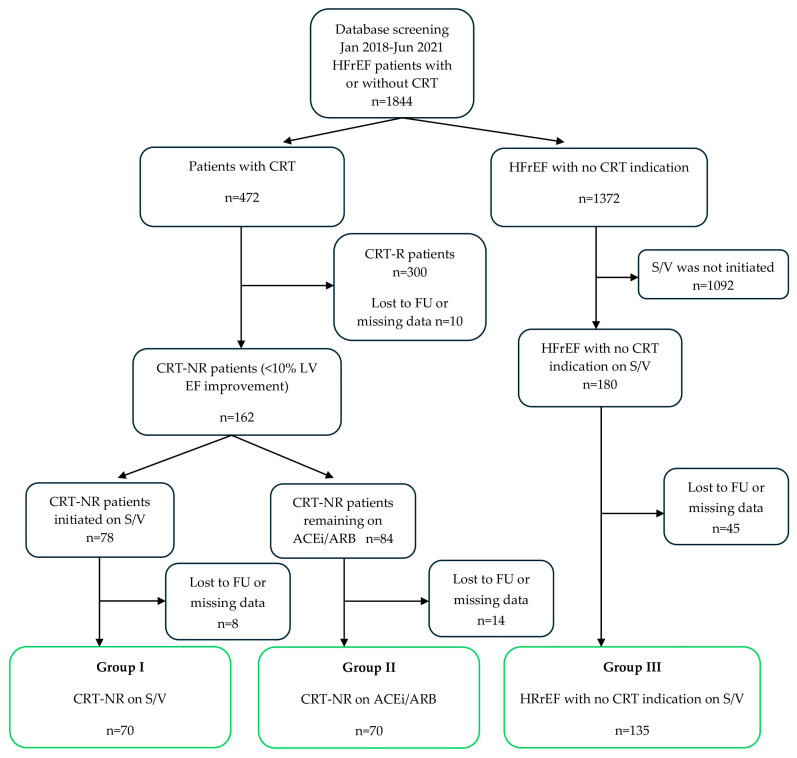
Flowchart showing patient recruitment and assignment into the 3 groups. S/V (sacubitril/valsartan); CRT-NR (nonresponder to cardiac resynchronization therapy); CRT-R (responder to cardiac resyncronization therapy); HFrEF (heart failure with reduced ejection fraction); HFH (heart failure hospitalization); HTx (heart transplantation); LVAD (left ventricular assist device); FU (follow-up).

The primary endpoint of the study was the composite of death from any cause, HTx, LVAD implantation, and heart failure hospitalization (HFH). HFH and the composite of death from any cause or the need for HTx or LVAD implantation were analyzed separately as secondary endpoints (Figure 1).

### 2.3. Statistical Analysis

Continuous variables are presented as median with interquartile range (IQR) unless otherwise specified. Normally distributed continuous variables are expressed as mean ± standard deviation (SD) and were compared using two-sample or paired Student’s *t*-tests, as appropriate. For non-normally distributed continuous variables, the Wilcoxon matched-pairs signed-rank test was applied. Normality was assessed using the Kolmogorov–Smirnov test. Comparisons between groups for continuous variables were performed using ANOVA. Survival analyses were performed using the Kaplan–Meier method, with differences between groups assessed by the log-rank test. Multivariable Cox proportional hazards regression models were constructed to evaluate associations with outcomes. A two-tailed *p* value < 0.05 was considered statistically significant. All statistical analyses were performed using Stata version 17 (StataCorp, College Station, TX, USA).

## 3. Results

### 3.1. Baseline Parameters

A total of 275 patients were enrolled in the study and assigned to one of the three groups: 70 patients in Group I, 70 patients in Group II, and 135 patients in Group III [12]. The baseline clinical characteristics of the three groups at the time of enrollment are depicted in Table 1. Patients in both CRT-NR groups (Group I and Group II) were older and presented with a higher prevalence of comorbidities as compared with the general heart failure with reduced ejection fraction cohort. There was no significant difference in the etiology of heart failure among the three groups. The majority of patients in all three groups were classified as NYHA functional class III, with median NT-proBNP levels (pg/mL) of 2058 (1041–4502), 1474 (655–5274), and 2223 (1233–4795) for Groups I, II, and III, respectively. 

All patients were receiving guideline-directed medical therapy for heart failure including ACEi, BB, and MRA at baseline. The median follow-up periods were 24 months, 23 months, and 24 months for Groups I, II, and III, respectively.

The ratios of CRT-D/CRT-P devices implanted at the discretion of the attending physician were 47.1%, 41.4%, and 59.2% in groups I, II, and III, respectively (*p* > 0.05). Appropriate ICD therapy (shock or ATP) was found in 9%, 15.3%, and 10% of patients in Groups I, II, and III, respectively (*p* > 0.05).

LV EF values measured 6–9 months after enrollment were 30 [25–35], 30 [24.7–35], and 29 [25–35], while NT-proBNP values 1121.55 [545–2541], 1986.3 [1025–3359], and 1123.09 [500.38–2651.27], in Groups I, II, and III, respectively.

### 3.2. Primary and Secondary Endpoint

During the entire follow-up period, the primary composite endpoint occurred in 27 out of 70 patients (38.57%) in Group I (HTx: 2; LVAD: 1), 43 out of 70 patients (61.42%) in Group II, and 60 out of 135 patients (44.42%) in Group III (HTx: 2). The differences between Groups I and II, as well as between Group II and III, reached the level of statistical significance, while the two groups on S/V (Group I and III) demonstrated similar outcomes (*p* = 0.465; Table 2).

**Figure 2:**
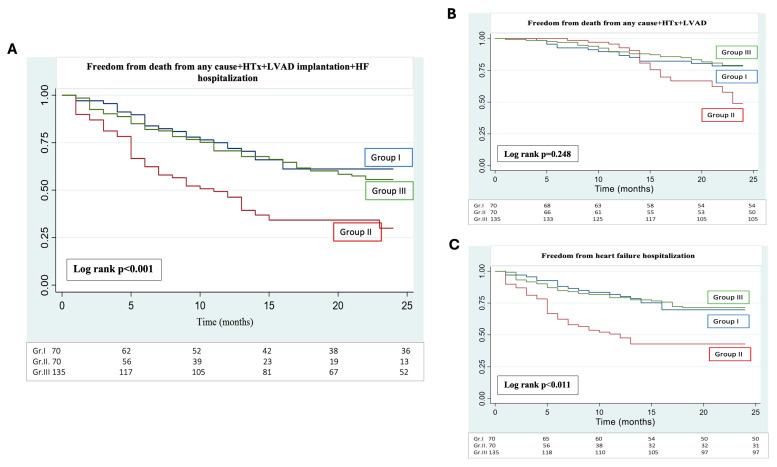
Primary endpoint: (**A**) freedom from all-cause mortality and heart failure hospitalization+heart transplantation (HTx)+left ventricular assist device implantation (LVAD)—primary composite endpoint—Kaplan–Meier analysis. Group I: CRT-NR+S/V; Group II: CRT-NR+ACEi/ARB; Group III: HFrEF without CRT+S/V. Secondary endpoints: (**B**) freedom from death from any cause—Kaplan–Meier estimates for secondary outcomes. HTX (heart transplantation); LVAD (left ventricular assist device); HF (heart failure); Group I: CRT-NR+S/V; Group II: CRT-NR+ACEi/ARB; Group III: HFrEF without CRT+S/V. (**C**) Freedom from heart failure hospitalization—Kaplan–Meier estimates for secondary outcomes. HTX (heart transplantation); LVAD (left ventricular assist device); HF (heart failure); Group I: CRT-NR+S/V; Group II: CRT-NR+ACEi/ARB; Group III: HFrEF without CRT+S/V.

Similarly, the secondary endpoint of HFH was significantly higher in Group II than in either Group I or Group III, while results were similar in the latter two cohorts of patients on S/V. Regarding the other secondary endpoint of death for any cause +LVAD+HTX (chi-squared test), significant differences were not found among the three groups. The statistical results as described above were confirmed with Kaplan–Meier analysis (Figure 2).

With multivariable Cox regression analysis for the primary endpoint adjusting for age, sex, and comorbidities including atrial fibrillation, diabetes mellitus, chronic kidney disease, and hypertension, CRT-NR patients on S/V treatment demonstrated a 64% relative risk reduction in the primary endpoint as compared with patients kept on ACEi/ARB therapy (hazard ratio [HR] 0.36 [95% confidence interval (CI) 0.21–0.60]; *p* < 0.001). Similarly, patients in Group I were less likely to be hospitalized for heart failure than patients in Group II. (HR 0.61 [95% CI 0.34–0.86]; *p* < 0.045). However, Cox regression analysis for the endpoint of mortality+HTx+LVAD implantation showed no significant difference between the two groups (HR 0.69 [95% CI 0.36–1.7]; *p* < 0.247).

LV EF and NT-proBNP values measured in Group I at the initiation of S/V and after 6–9 months on therapy in patients with versus without a composite endpoint event during long-term follow-up are displayed in Table 3. Both LV EF and NT-proBNP demonstrated significant improvements in patients with no composite endpoint, while those with an event also showed a marginally significant improvement in NT-proBNP, but not in LV EF. At least a 5% increase in LV EF was measured in 39 out of the 70 patients (55.7%) and 33 of them (91.7%) remained free of a composite endpoint until the end of follow-up, while 15 out of the 31 patients (48.4%) with no improvement (no or less than 5% increase in LV EF) did have an event. NT-proBNP levels on S/V were below 1000 pg/mL in 27 out of 70 patients (38.6%), and 24 of them (88.9%) had composite-endpoint-free survival.

## 4. Discussion

Herein, the clinical outcomes of CRT-NR patients initiated on S/V were compared with those remaining on ACEi/ARB. In addition, we included a third group of HFrEF patients with no indication for CRT who were also switched to S/V as a replacement for ACEi/ARB. The rationale for this double comparison design was to investigate if therapy escalation with the addition of S/V could improve the outcome in CRT-NRs and to test whether the long-term outcome was comparable to what can be achieved in a general patient cohort on S/V treatment. The occurrence of the composite endpoint (all-cause mortality/HTx/LVAD implantation and HFH) throughout the whole follow-up demonstrated a significant benefit of S/V treatment in CRT-NR patients as compared with those on ACEi/ARB and was comparable to the endpoint events observed in the general HFrEF cohort treated with S/V. The improvement in the composite endpoint was driven by the lower occurrence of HFH with S/V therapy as compared with ACEi/ARB, while between-group differences in all-cause mortality/HTx/LVAD implantation did not reach the level of statistical significance at this sample size. 

The first trial demonstrating a significant clinical benefit of S/V as compared with enalapril was the PARADIGM-HF study, which showed a 20% relative risk reduction (RRR) for the composite endpoint, 21% RRR for HFH, and 16% for all-cause mortality [8]. Importantly, only 7% of patients had previous CRT in this trial, with no information disclosed on the response to device therapy. In our research, the switch to S/V treatment in CRT-NRs demonstrated a robust 64% RRR for the primary composite endpoint and a 39% RRR for HFH. Total mortality/HTx/LVAD rates were 20% in both groups (I-III) on S/V and 27.14% on ACEi/ARB (Group II; *p*: 0.468). Notably, all-cause mortality rates in the PARADIGM-HF trial were 17.0% versus 19.8% in the S/V versus enalapril treatment groups, respectively, and the 16% RRR was statistically significant (*p* < 0.001) in a much larger sample size than in our research. 

As reported earlier [13], in the same patient cohort we analyzed herein, LV reverse remodeling, as indicated by an increase in left ventricular ejection fraction and reductions in both left ventricular end-systolic diameter (LVESD) and NT-proBNP levels, was observed in the CRT nonresponder group after receiving S/V treatment for a duration of 6 to 9 months, similarly to the general cohort of patients with heart failure with reduced ejection fraction (HFrEF). A comparison confined to patients in Group I who did and did not have a primary composite endpoint throughout the whole follow-up demonstrated a significant correlation between reverse remodeling detected earlier (6–9 months) after the initiation of S/V therapy and the longer-time (~2 year) outcome: significantly higher LV EF and lower levels of NT-proBNP were detected in patients free of a composite endpoint event. This finding is in line with previous reports, which demonstrated the significant correlation between LV reverse remodeling initiated by medical or device therapy and the long-term outcome in HFrEF patients [14]. According to our analysis, a minimum of a 5% increase in LV EF predicted a 91.7% event-free survival, while a reduction in NT-proBNP level below 1000 pg/mL predicted an 88.9% event-free survival, until the end of follow-up in CRT-NRs.

A unique aspect of our research design was the inclusion of a general HFrEF cohort for comparison with CRT-NR patients. Although CRT-NRs presented with a less favorable clinical profile, including older age and higher prevalence of comorbidities such as diabetes mellitus, atrial fibrillation, and chronic kidney disease, S/V therapy resulted in comparable outcome benefit in the two patient groups that received it (Groups I and III). Notably, baseline LV function and NT-proBNP levels at the time of S/V initiation were comparable in the two cohorts, suggesting that myocardium contractile reserve might be a more significant prognosticator in HFrEF and underscoring the clinical value of this therapy.

We have been able to find only a few reports with outcome data in the specific cohort of CRT-NR patients after the initiation of S/V as a replacement for ACEi/ARB treatment [15,16,17]. In a prospective observational study including 190 patients who were classified as nonresponders at least 12 months after device implantation, S/V therapy was initiated for at least another 12 months [14]. Significant improvements in functional class and HFH were observed in those 37 patients, who demonstrated a >15% decrease in LV ESV. In the RESINA study, quality of life and HFH improved in 35 CRT-NRs after switching from ACEi/ARB to ARNI medication [15]. Further, in a retrospective analysis, Chun et al. compared the outcome of CRT-NRs who remained on ACEi/ARB (28 patients) with those who were switched to S/V (22 patients) during a 30-month median follow-up [16]. The sum of all-cause mortality, HTx, and LVAD implantation was significantly lower, while HFH showed a tendency for improvement (*p* = 0.068), on S/V medication. 

### 4.1. Clinical Implications

According to recent guidelines recommendations, medical treatment of all HFrEF patients should include S/V as part of the initial therapy or as a replacement of ACEi/ARB medication in the absence of significant improvement [6,7] to improve quality of life, survival, and rate of HFH. Data from the literature also indicate that S/V may provide significant improvement in HFrEF across a wide range of LV function and clinical status [10,18]. Results from prospective randomized studies on the efficacy and the clinical value of replacing ACEi/ARB medication with S/V in the specific cohort of CRT-NRs are not available. In our view, the initiation of a randomized trial as of today would raise ethical issues, considering the robustness of data on the advantage of ARNI in HFrEF patients, especially in those showing no or only moderate improvement. The results of observational studies, including ours, in the specific cohort of CRT-NRs should be viewed as pieces of evidence strongly supporting this strategy. Notably, the outcome of these patients is strikingly poor, with survival rates less than 50% without the need for a mechanical assist device or cardiac transplantation within five years postimplantation [19]. In addition, data from the ADVANCE CRT registry indicate that many CRT-NR patients are managed passively in clinical practice with no effort to maximize available therapeutic options, including medical therapy [2]. Our results support the initiation of S/V in this “forgotten” cohort of HF patients.

### 4.2. Limitations

This was a single-center observational study that involved a limited number of patients. Nonetheless, the population size in our analysis was still greater than that reported in the limited number of publications on the efficacy of S/V in patients who are CRT-NRs. With the double comparison design of our research, we were able to demonstrate the outcome benefit of S/V in the specific cohort of CRT-NRs, which was comparable to what can be expected in a general HF patient population after the initiation of S/V. Death from any cause was calculated instead of cardiovascular mortality in order to avoid the potential bias from misclassification of death. Although SGLT2-inhibitors are now recommended as the fourth pillar of medical treatment in all HF patients [6,7], this medication was not available during the time of our study. 

Further, patients demonstrating cardiac remodeling after CRT were excluded from this study focusing on CRTNR. However, CRT responders might also benefit from switching ACEi/ARB treatment to S/V. This clinically relevant issue should be the subject of future research. 

## 5. Conclusions

Our research demonstrates the benefit of therapy escalation from ACEi/ARB to S/V in patients with no adequate response to CRT. The composite endpoint of all-cause mortality, HTx, the need for LVAD implantation, and HFH was similar in CRT-NR patients on SV therapy and in general HFrEF patients who were also started on S/V, and significantly better in both groups taking S/V than in CRT-NR patients remaining on ACEi/ARB. The positive changes were mainly driven by the significant improvement in HFH in both patient groups on S/V therapy. These data support the use of S/V as a replacement for ACEi/ARB in CRTNR patients. 

## Figures and Tables

**Table 1:** Baseline characteristics of the 3 patient groups at the time of enrollment. CKD (chronic kidney disease); MR antagonists (mineralocorticoide receptor antagonists); ARBs (angiotensin receptor blockers); LV EF (left ventricular ejection fraction); NT-proBNP (N-terminal pro-B-type natriuretic peptide); CAD (coronary artery disease).

	Group I (n = 70)	Group II (n = 70)	Group III (n = 135)	*p*-Value
Age (years), mean ± SD	66.1 ± 9.1	65.5 ± 11.3	62.4 ± 11.3	**0.018**
Female, n (%)	9 (12.9%)	9 (12.9%)	31 (22.9%)	0.128
Body weight, (kg) mean ± SD	90.9 ± 18.8	89.1 ± 20.9	92.3 ± 23.6	0.669
CAD, n (%)	33 (47.2%)	26 (37.1%)	66 (48.9%)	0.264
Hypertension, n (%)	67 (97.7%)	49 (70%)	89 (65.9%)	**˂0.001**
Diabetes, n (%)	42 (60%)	35 (50%)	48 (35.6%)	**0.003**
CKD, n (%)	26 (37.2%)	31 (44.2%)	36 (26.7%)	**0.033**
Atrial fibrillation, n (%)	39 (55.7%)	23 (32.8%)	46 (34.1%)	**0.006**
Dyslipidemia, n (%)	64 (91.4%)	36 (51.4%)	74 (54.9%)	**0.001**
LV EF, median, IQR, %	25 [20–29.2]	28.5 [25–35]	27 [22–30]	**0.005**
LVESD, median, IQR, mm	55 [48–62]	50 [46–61]	55 [49–64]	**0.016**
NT-proBNP, median IQR, pg/mL	2058 [1041–4502]	1474 [655–5274]	2223 [1233–4795]	**<0.001**
Pharmacological Therapy				
ACE inhibitors/ARBs, n (%)	70 (100%)	68 (97.1%)	135 (100%)	0.052
Beta blockers, n (%)	67 (95.7%)	68 (97.1%)	127 (94.1%)	0.661
MR antagonists, n (%)	65 (92.8%)	67 (95.7%)	124 (91.8%)	0.355

**Table 2:** Primary and secondary endpoints during follow-up after S/V therapy initiation in the three patient groups. Data are presented as n (%). * *p* values were obtained by Chi-squared test. ** *p* values were obtained by proportion Z-test (pairwise comparisons). Group I: CRT-NR+S/V; Group II: CRT-NR+ACEi/ARB; Group III: HFrEF without CRT+S/V. HTx (heart transplantation); LVAD (left ventricular assist device).

Endpoints	Group I	Group II	Group III	*p* Value (Group I vs. II vs. III) * Chi-Squared	*p* Value (Group I vs. Group II) ** Proportion Z-Test	*p* Value (Group I vs. Group III) **	*p* Value (Group II vs. Group III) **
Primary composite endpoint (death from any cause+HTx+LVAD+ heart failure hospitalization (n, %)	27/70 (38.57%)	43/70 (61.42%)	60/135 (44.44%)	0.010	0.005	0.465	0.012
Death from any cause+HTx+LVAD implantation (n, %)	14/70 (20%)	19/70 (27.14%)	27/135 (20%)	0.468	0.341	0.962	0.245
Heart failure hospitalization (n, %)	19/70 (27.14%)	38/70 (54.28%)	36/135 (26.66%)	<0.001	0.001	0.896	<0.001

**Table 3:** LV EF and NT-proBNP values in Group I at the initiation of sacubitril/valsartan (S/V) and after 6–9 months on therapy in patients with or without a composite endpoint event during follow-up. IQR (interquartile range, 25th–75th percentile).

	LV EF at Initiation of SV (%, Median and IQR)	LV EF 6–9 Months After Initiation of SV (%, Median and IQR)	*p*-Value	NT-proBNP at Initiation of SV (pg/mL, Median and IQR)	NT-proBNP 6–9 Months After Initiation of SV (pg/mL, Median and IQR)	*p*-Value
No composite endpoint event	25 (20–29.7)	30 (25–36)	<0.001	1556 (1030–4548)	989.7 (461–2046)	<0.001
Composite endpoint event	24 (20–28)	27 (19.5–30)	0.601	2559 (2273–4869)	1723 (1102–3193)	0.030

## Data Availability

Anonymized data are available upon reasonable request (Debrecen University Hospital IT System: UD Med).

## Abbreviations

The following abbreviations are used in this manuscript:

ARNI	angiotensin receptor neprilysin inhibitor
HF	heart failure
HFrEF	heart failure with reduced ejection fraction
S/V	sacubitril/valsartan
CRT	cardiac resynchronization therapy
CRT-NR	CRT nonresponder
CRT-NRs	CRT nonresponders
ACEi/ARB	angiotensin converting enzyme inhibitors/angiotensin receptor blockers
CRT-P	cardiac resynchronization therapy–pacemaker
CRT-D	cardiac resynchronization therapy–defibrillator
NT-proBNP	N-terminal pro-B-type natriuretic peptide
HTx	heart transplantation
LVAD	left ventricular assist device
HFH	heart failure hospitalization
LV EF	left ventricular ejection fraction
LV ESVi	left ventricular end-systolic volume index
LV ESV	left ventricular end-systolic volume
LV ESD	left ventricular end-systolic diameter
ESC	European Society of Cardiology
ACC	American College of Cardiology
